# Segmental Humeral Head Reconstruction in Patients with Chronic Locked Posterior Shoulder Dislocation

**DOI:** 10.3390/medicina59101736

**Published:** 2023-09-28

**Authors:** Srđan Ninković, Vukadin Milankov, Milan Tošić, Milan Majkić, Branko Baljak, Milan Milinkov, Mirko Obradović

**Affiliations:** 1 Clinic for Orthopedic Surgery and Traumatology, Clinical Center of Vojvodina, 21000 Novi Sad, Serbia; 911025d22@mf.uns.ac.rs (M.T.); milan.majkic@yahoo.com (M.M.); bane95ns@gmail.com (B.B.); 014293@mf.uns.ac.rs (M.M.); 1141d16@mf.uns.ac.rs (M.O.); 2 Faculty of Medicine, Department of Surgery, University of Novi Sad, 21000 Novi Sad, Serbia; vukadin.milankov@mf.uns.ac.rs; 3 Institute for Children and Youth Health Care of Vojvodina, 21000 Novi Sad, Serbia

**Keywords:** shoulder, humeral head, shoulder dislocation, shoulder fractures, allografts

## Abstract

*Background and Objectives*: The goal of this study was to evaluate the functional outcomes of patient treatment using an allograft after chronic locked posterior shoulder dislocation associated with a bony defect of the upper edge of the humerus that involves 25–50% of the articular surfaces. *Materials and Methods*: A total of 20 patients were included in this study. Electrocution was the cause of injury in eight patients; in ten patients, the cause was direct trauma; and in two patients, the cause of injury was a fall due to hypoglycemic coma. A standard deltoid pectoral approach was used and a fresh-frozen osteochondral allograft of the femoral condyle was applied. In evaluating the results, Constant’s scoring scale was used. *Results*: The average value of Constant’s point scale for the operated shoulder is 84.14 points. This result is good according to the average value of Constant’s point scale. *Conclusions*: Patients with locked chronic posterior dislocation in combination with a bony defect of the humeral head that covers 25–50% of the articular surface, in our opinion, should be treated using bone allografts rather than non-anatomical reconstruction methods.

## 1. Introduction

Posterior shoulder dislocation, although relatively uncommon, presents a significant clinical challenge in the field of orthopedics. It is estimated that nearly two-thirds of cases go unrecognized initially [1]. This intriguing condition typically arises due to a variety of factors, including high-intensity forces, electrical shocks, or epileptic seizures, all of which culminate in an intricate interplay of muscle contractions within the shoulder joint. The overwhelming action of the internal rotator muscles, such as the pectoralis major, latissimus dorsi, subscapularis and teres major, exerts tremendous force, effectively driving the shoulder into an internally rotated position. This cascade of events ultimately leads to the humeral head’s posterior displacement, giving rise to the challenging clinical scenario of posterior shoulder dislocation [2]. Importantly, this traumatic event often brings with it a distinctive bone lesion, a hallmark of posterior shoulder dislocation—an impressive fracture of the anteromedial aspect of the articular surface of the humeral head. This fracture, commonly referred to as a “reverse Hill–Sachs lesion”, adds further complexity to the management of the condition, warranting a tailored approach to its treatment [3].

The therapeutic strategy for posterior shoulder dislocation significantly depends on the extent of the associated bone defect within the humeral head. A range of treatment modalities is at the disposal of orthopedic surgeons. When confronted with bone defects involving 25% to 50% of the articular surface of the humeral head, anatomical reconstruction techniques emerge as a crucial intervention. These techniques encompass a spectrum of strategies, including transferring the attachment of the subscapular muscle tendon to the site of the defect [4], employing the modified McLaughlin technique [5], or conducting a rotation osteotomy of the proximal humerus [6].

Regarding anatomical procedures, there are several advantages and disadvantages. The advantages of anatomical procedures include the restauration of the native spherical contour of the humeral head, the maintenance of normal range of motion of the shoulder joint a biomechanically stable joint and the possibility of future joint replacement (arthroplasty procedures). On the contrary, the disadvantages are graft resorption, hardware impingement, disease transmission and delay or nonunion.

However, the choice of treatment approach is not one-size-fits-all. Each patient presents a unique clinical scenario, and the orthopedic surgeon must carefully assess the specifics of the case. The ultimate goal is to restore shoulder stability and function while minimizing the risk of complications. On the other end of the treatment spectrum, when faced with more extensive bone defects affecting over 50% of the humeral head’s articular surface, the treatment paradigm shifts towards arthroplasty procedures. In such a cases, hemiarthroplasty or total shoulder joint arthroplasty may come into consideration [7].

The decision between non-anatomical and anatomical reconstruction is not made lightly. Orthopedic surgeons must carefully weigh the benefits and risks of each approach, taking into account the patient’s age, overall health, and functional requirements. The goal is to provide the most appropriate and effective treatment to address the unique challenges posed by posterior shoulder dislocation.

The aim of this study was to evaluate the results of the treatment of locked posterior shoulder dislocation associated with reverse Hill–Sachs lesion using anatomical reconstruction procedures in our institution. Our hypothesis was that osteochondral allograft transplantation is a useful treatment option that gives favorable clinical outcomes for reverse Hill–Sachs lesion including 25–50% defect of the humeral head articular surface. We also have in mind the fact that there is no gold standard (algorithm) in the treatment of the mentioned injuries.

## 2. Materials and Methods

A retrospective study was conducted after receiving approval from the Ethics Committee of the University Clinical Center of Vojvodina. The study involved 20 patients over a seven-year period. The inclusion criteria were as follows: (1) patients older than 18; (2) patients diagnosed with locked posterior shoulder dislocation associated with reverse Hill–Sachs lesion (involving humeral head articular surface defect of 25 to 50%) during the mentioned seven-year period; (3) patients who had undergone adequately performed X-ray and CT diagnostics preoperatively. The exclusion criteria were as follows: (1) patients with intensive damage to the glenoid fossa; (2) patients with associated injuries.

**Figure 1:**
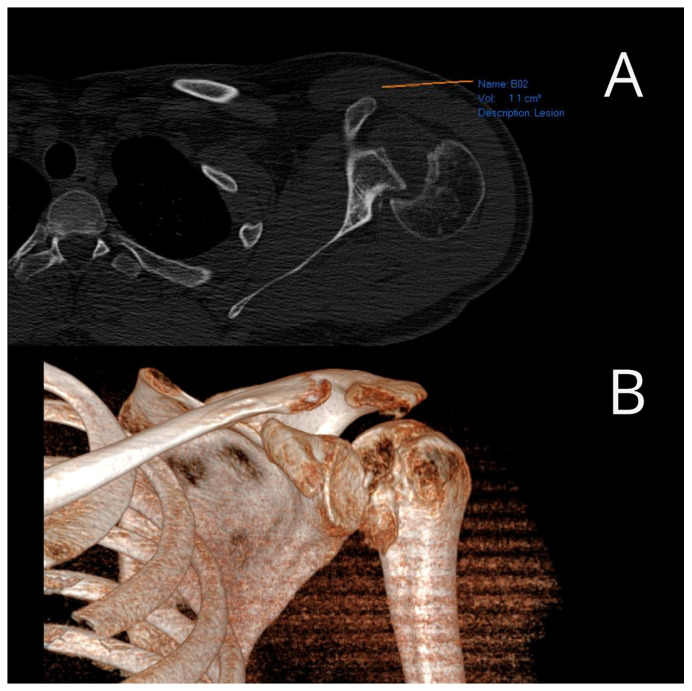
Preoperative axial (**A**) and three-dimensional (**B**) CT image of a reverse Hill–Sachs lesion (photo from the personal archive of intraoperative photos of the author).

All examinees signed their informed consent. Patients underwent surgery at the Clinic for Orthopedic Surgery and Traumatology of the University Clinical Center of Vojvodina. Electrocution was the cause of injury in eight patients, direct trauma in ten patients and a fall due to hypoglycemic coma in two patients. There was no positive family history among the patients, and all injuries were unilateral. Eighteen patients had injured their dominant hand. Prior to the surgery, all patients underwent computed tomography (CT) scans to quantify the reverse Hill–Sachs lesions (Figure 1).

Following the confirmation of the diagnosis through traditional radiography, all patients underwent computed tomography (CT) scans. The dimensions of the defect in the humeral head were gauged using the preoperative CT scan while the head was dislocated posteriorly. A circular marker was placed over the humeral head in the CT scan taken at or just below the coracoid level. The cartilage angle was determined by two lines extending from the center of the circle to the cartilage immediately adjacent to the lesser tuberosity and the posterior end of the cartilage adjacent to the infraspinatus insertion. Another angle, referred to as the defect angle, was calculated by measuring the angles formed by connecting the anterior limit and posterior limit of the defect with the center of the humeral head. The size of the humeral head defect was estimated by determining what percentage of the cartilage angle the defect angle represented. A posterior glenoid rim defect that exceeded half of the maximum anteroposterior diameter was considered clinically significant.

The surgical procedures were performed under general anesthesia with the patients in a beach chair position, allowing anterior and posterior access to the shoulder, as well as intraoperative X-ray control. A standard deltoid–pectoral approach was used for humeral head reconstruction. An osteotomy of a portion of the small tubercle of the humerus, along with the muscular attachment of the subscapular muscle, was performed. We took care to avoid injury to the anterior circumflex vessels and protected the arcuate artery lateral to the bicipital groove. Lateral capsulotomy and the excision of any joint scar tissue were also conducted. The reduction was carefully performed to avoid significant damage to the humeral head or the glenoid fossa. We performed the reduction by placing the hand in internal rotation and pressing from the back on the humeral head using a bone hook placed in the bone defect of the humeral head. After reduction, the joint was stabilized in a position of neutral rotation. We noticed that the posterior capsule was stretched, while other rotator cuff muscles (m. supraspinatus, m. infraspinatus and m. teres minor), which are attached to the greater tubercle of the humerus, were not damaged during either the surgical procedure or joint reduction.

**Figure 2:**
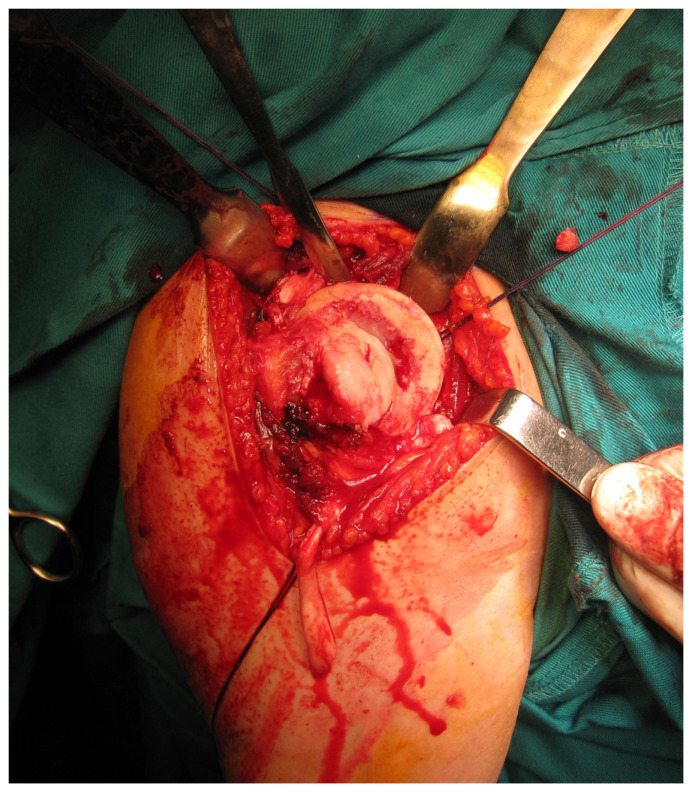
Intraoperative presentation of a reverse Hill–Sachs lesion (photo from the personal archive of intraoperative photos of the author).

**Figure 3:**
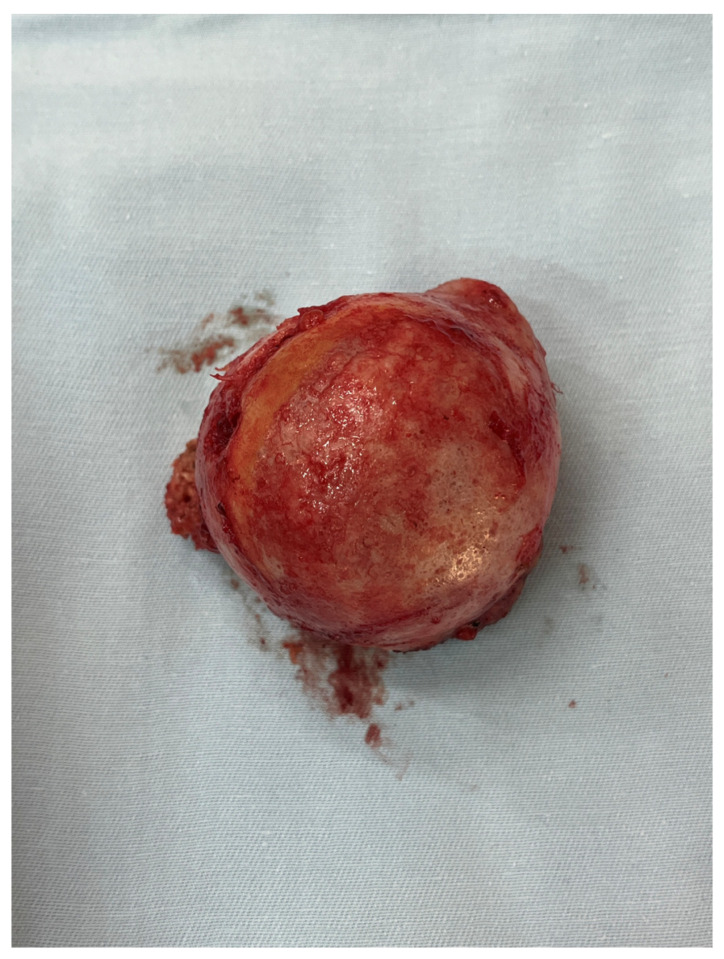
Osteochondral femoral allograft (photo from the personal archive of intraoperative photos of the author).

**Figure 4:**
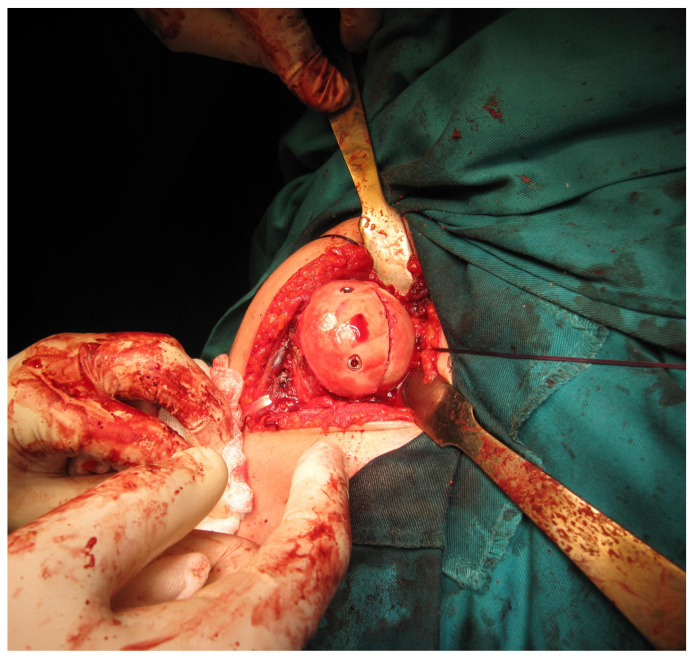
Illustration of fixed allograft with two cancellous screws (photo from the personal archive of intraoperative photos of the author).

All femoral condyle osteochondral allografts were fresh-frozen and stored at −80 °C. They were tested for bacterial contamination, as well as serologically for hepatitis (A, B and C) and human immunodeficiency virus. On the day of surgery, the osteochondral grafts were thawed and submerged in 300 mL of saline solution with five ampoules of 120 mg gentamycin. Before placement, the grafts were washed with 500 mL of pure physiological solution. An oscillating saw was used to prepare the humeral head defect site for the osteochondral allograft. The dimensions of the lesion were carefully measured, and a similarly sized allograft was modeled accordingly (Figure 2 and Figure 3). Tenodesis of the long head of the bicep tendon was performed in all patients. Fixation of the allograft to the humeral head was achieved using two cancellous screws in four patients, while two Herbert screws [8] were used in the remaining sixteen patients, all placed subchondrally (Figure 4). After the shoulder joint was reduced into place, the reinsertion of the m. subscapularis was performed in its anatomical position (because osteotomy of the lesser tubercle was performed during the procedure). During the immobilization period, the m. subscapularis healed in its anatomical position. The shoulder joint capsule was sutured with absorbable sutures and the wound was closed in layers.

After the operative treatment, the arm was immobilized in a neutral position for six weeks. Physical therapy started on the first day, with the immobilization being removed three times a day to perform passive exercises, focusing on flexion and external rotation. Internal rotation was not permitted for six weeks. Active exercises began six weeks post-surgery, followed by active resistance exercises after 12 weeks. Physiotherapy was continued for the next six months, and no complications regarding wound healing or neurovascular injury were reported.

Evaluation of the results was carried out two years after the surgical procedure using the Constant shoulder score [9], which combines an objective physical examination (65 points) with a subjective assessment from the patients (35 points).

## 3. Results

### Main Results

The average age of the patients was 60.2 ± 14 years (ranging from 36 to 70 years). The sex distribution of the sample consisted of 15 (75%) men and 5 (25%) women.

The mean time from injury to diagnosis was 205 days (ranging from 35 to 420 days). The time period from injury to surgical treatment was 220.8 days (ranging from 42 to 434 days). Following surgical treatment, two patients (10%) reported experiencing mild pain, while eighteen patients (90%) stated that they had no pain in the operated shoulder. Eighteen patients (90%) were able to perform all activities of daily life smoothly, one patient (5%) experienced occasional limitations and another patient (5%) reported constant limitations in performing daily activities. Follow-up time was two years postoperatively.

**Figure 5:**
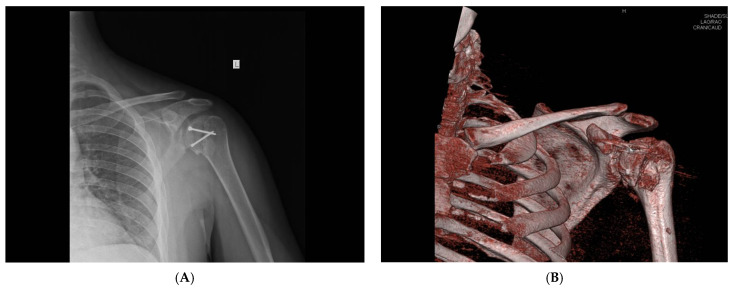
Postoperative X-ray (**A**) and three-dimensional CT view (**B**) of the operated shoulder (photo from the personal archive of intraoperative photos of the author).

The average value of Constant’s point scale for the operated shoulder was 84.14 (ranging from 50 to 93) (Table 1), indicating a good result. Postoperatively, a follow-up X-ray and CT scan were performed, which demonstrated the appropriate positioning of the allograft in all twenty patients (Figure 5).

No shoulder joint instability was observed in any patient after surgical treatment. Avascular necrosis of the humeral head occurred in one patient (5%) following surgery. There were no early or late signs of infection, pseudarthrosis, redislocation, neurovascular injuries or collapse of the implemented bone allograft in any patient.

## 4. Discussion

Posterior shoulder dislocation, constituting a mere 2–4% of all shoulder joint dislocations, is a distinctly rare injury in the realm of orthopedics. This rarity has contributed to its often elusive nature, with a staggering two-thirds of these injuries eluding initial detection during clinical examination [10,11]. This diagnostic challenge is exacerbated by the fact that posterior shoulder dislocation shares striking clinical similarities with adhesive capsulitis, colloquially known as “frozen shoulder” [12]. The hallmark of this shared clinical presentation is a notable deficit in external rotation, which affects both active and passive movements.

However, while adhesive capsulitis typically develops gradually and without a clear precipitating event, posterior shoulder dislocation emerges abruptly, often following trauma or one of the aforementioned causes of injury. This stark contrast in etiology underscores the critical importance of a thorough patient history and examination to distinguish between the two conditions.

Furthermore, what sets posterior shoulder dislocation apart is the often accompanying and strikingly conspicuous fracture of the anteromedial aspect of the articular surface of the humeral head. This fracture, referred to as the “reverse Hill–Sachs lesion”, serves as a distinctive marker of posterior shoulder dislocation. The presence of this fracture not only aids in the diagnosis but also adds an additional layer of complexity to the management of this condition, necessitating a tailored approach to treatment [3]. Understanding these nuances is paramount for orthopedic clinicians when faced with patients presenting with shoulder pain and restricted external rotation, enabling accurate diagnosis and effective intervention to restore shoulder function and stability.

Our comprehensive study focused on a cohort of 20 patients who presented with posterior shoulder dislocation accompanied by a notable fracture of the anteromedial aspect of the humeral head. These individuals underwent a carefully orchestrated treatment protocol involving the implantation of a bone allograft sourced from the esteemed bone bank of the Clinic for Orthopedic Surgery and Traumatology at the University Clinical Center of Vojvodina. The primary objective of this intervention was to achieve anatomical reconstruction of the humeral head, addressing not only the dislocation but also the concomitant bone defect. This approach to posterior shoulder dislocation aligns with the methodologies described by several esteemed authors in the field [11,13,14,15]. These experts have previously explored the application of allograft materials in cases where the bone defect encompasses 25% to 50% of the joint surface. The overarching goal of such interventions is to restore the intricate architecture of the shoulder joint, ensuring both its stability and function.

Gerber and Lambert [11] presented a series of four patients with a bone defect of the humeral head covering 40–50% of the articular surface, treated with an allograft. They reported that three patients had a good result, while one patient had a poor result. In a study by Diklić et al. [13], which included 13 patients, all patients underwent surgical treatment using an allograft. After reconstruction, 12 patients achieved a good result, while one patient experienced avascular necrosis of the humeral head, leading to allograft collapse. Murphy et al. [14] presented a study involving five patients with posterior shoulder dislocation combined with a reverse Hill–Sachs lesion [3]. Following reconstruction, four patients had a good result, while one patient developed secondary osteoarthritis of the shoulder joint. Martinez et al. [15] conducted research involving six patients with posterior shoulder dislocation associated with a bone defect of the upper edge of the humerus, treated with an allograft. Out of the six patients, three had a good result, while three had a poor result. Despite the fact that according to Constant’s scale, 50% of patients have poor result, none of them underwent shoulder arthroplasty, which indicates the success of treating this injury using an allograft.

We compared the obtained range-of-motion values of the operated shoulder with those reported in other studies [11,13,14,15]. The lower degree of external rotation in our study can be attributed to the older age of the patients and the prolonged period from the moment of injury to surgical treatment, resulting from inadequate treatment prior to their arrival at our institution. Nevertheless, the patients expressed satisfaction with their subjective feeling of recovery, and their achieved range of motion, without any pain, is sufficient for normal activities of daily life. It is important to emphasize that most movements in daily activities occur within the plane of the scapula, involving flexion and abduction up to 90°. In our study, CT scans were performed on all patients. This diagnostic method is essential for quantifying the reverse Hill–Sachs [3] lesion and evaluating the incorporation of the bone allograft, consistent with findings from the literature [13,14,15,16,17]. Avascular necrosis of the humeral head occurred in one patient (5%) in our study. In the study published by Diklić et al. [13], one out of thirteen patients developed avascular necrosis of the humeral head, resulting in allograft collapse. Gerber and Lambert [11] reported that avascular necrosis of the humeral head occurred in one patient in their study. Martinez et al. [15] reported that allograft collapse occurred in two patients. They attributed the worse results to the longer follow-up period, which increases the likelihood of post-traumatic osteoarthritis and allograft collapse over time. In the research conducted by Ruttershoff et al. [18], one subject did not achieve glenohumeral joint stability after segmental reconstruction using an allograft, necessitating the placement of a tricortical autograft from the iliac crest on the posterior edge of the glenoid. In our study, full stability of the glenohumeral joint was achieved following segmental reconstruction of the humeral head.

The management of chronic locked posterior dislocation of the shoulder can encompass various approaches, including the utilization of allografts, the application of arthroplasty procedures, or the utilization of autografts harvested from the contralateral shoulder, albeit exclusively in cases of bilateral shoulder dislocations. We posit that the employment of allografts in the context of anatomical procedures for addressing chronic locked posterior shoulder dislocation represents a more efficacious strategy when contrasted with the initial recourse to non-anatomical techniques, specifically arthroplasty procedures, while still allowing for the potential utilization of select non-anatomical prosthetic techniques. It is noteworthy that none of our patients had a humeral head articular surface defect exceeding 50% of its total area. In such instances, the preference leans toward the adoption of anatomical procedures over the primary implementation of prosthetic interventions. Additionally, studies have shown that in such cases, the use of anatomical procedures is associated with higher average Constant’s scores compared to arthroplastic procedures [13,19,20,21]. In recent years, numerous published manuscripts have delved into the realm of simplifying a complex surgical challenge. This particular problem lacks a universally accepted gold standard or a well-established treatment algorithm. The primary approach taken in these manuscripts involves the simplification of the surgical technique itself. However, it is important to note that these efforts are still relatively nascent and have not undergone extensive time-tested evaluation, unlike the prior utilization of allografts in this context [22,23].

Drawing upon the comprehensive dataset gleaned from our study and the insights garnered from other notable studies [11,13,15], it becomes increasingly evident that the treatment of chronic locked posterior dislocation of the shoulder joint, particularly when coupled with a concurrent bony defect of the humeral head, exhibits a commendable track record of success with a notably low incidence of complications. This empirical evidence underscores the viability and efficacy of employing allografts in addressing this intricate orthopedic challenge. The utilization of allograft materials serves as a pivotal component in achieving favorable outcomes by facilitating the restoration of both the structural integrity and functional capacity of the shoulder joint.

However, it is imperative to acknowledge that our study, while contributing valuable insights, bears certain inherent limitations. Foremost among these limitations is the relatively modest sample size upon which our findings are based and relatively short follow up time. The rarity of chronic locked posterior shoulder dislocation, especially in conjunction with a bony defect, poses a significant challenge in assembling a substantial cohort of subjects for analysis. This rarity is, in itself, a testament to the infrequency of this clinical presentation, further accentuating the need for comprehensive, multi-center collaborations to enhance our understanding.

Additionally, we must acknowledge the subjective nature of patient questionnaire responses, which formed an integral part of our data collection. Patient-reported outcomes are invaluable but may be influenced by various factors, including individual perceptions and personal experiences.

While our study provides valuable insights into the treatment of chronic locked posterior shoulder dislocation, there are several promising avenues for future research. Further investigations can explore the long-term outcomes and durability of anatomical reconstruction using allografts, providing a deeper understanding of the procedure’s sustainability and potential late complications. Additionally, prospective studies with larger cohorts can delve into the influence of patient-specific factors, such as age, comorbidities and preoperative functional status, on surgical outcomes. Understanding how these variables may impact the success of allograft-based reconstruction can guide patient selection and refine treatment strategies. Lastly, collaborative research efforts could investigate innovative techniques or materials for reconstruction, paving the way for advancements in the field of shoulder surgery. These future research directions hold the potential to further improve the management of chronic locked posterior shoulder dislocation and enhance patient outcomes. In conclusion, it should not be forgotten that the use of allografts in the treatment of posterior shoulder dislocations is a time-tested method. Today, it is accompanied by a straightforward surgical technique that has been perfected over the years, along with advancements in surgical instrumentation. Additionally, it has demonstrated favorable functional outcomes and a minimal number of perioperative complications, as our study has also shown. Based on the aforementioned facts, we assert that allografts should be regarded as the first choice for the treatment of these injuries.

## 5. Conclusions

In our comprehensive study, we have delved into the intricate management of chronic locked posterior shoulder dislocation accompanied by a bony defect affecting 25% to 50% of the articular surface of the humeral head. Through our rigorous investigation, we have arrived at a conclusive recommendation that carries significant implications for the treatment of these challenging cases.

Patients presenting with this specific condition are often faced with a complex decision-making process regarding their treatment. Our findings strongly advocate for a treatment paradigm that prioritizes the utilization of bone allografts over non-anatomical reconstruction methods. This shift in approach is rooted in our observation that bone allografts offer distinct advantages in terms of long-term stability and functional outcomes.

While non-anatomical reconstruction methods have been historically employed, they may not provide the same degree of anatomical restoration and long-term durability as allograft-based approaches. By opting for a bone allograft, clinicians can achieve a more anatomically sound reconstruction, which is crucial for maintaining the intricate biomechanics of the shoulder joint. This, in turn, promotes better long-term stability and function for our patients.

However, it is important to acknowledge that even with the utilization of bone allografts, there may be cases where integration and stability are not fully achieved, and the graft may collapse over time. In such instances, we propose a contingency plan that involves prosthetic replacement of the joint. The advantage of this approach lies in its ability to maintain the anatomical relationships within the shoulder joint. The prosthetic replacement not only preserves the natural alignment but also ensures sufficient stability, functionality and pain reduction for our patients. This adaptability in treatment options underscores our commitment to delivering the best possible outcomes for each individual case.

In conclusion, our study’s recommendations are driven by a comprehensive analysis of functional outcomes and patient experiences. We believe that by advocating for the use of bone allografts and considering prosthetic joint replacement as a viable backup plan, we are taking a significant step towards enhancing the quality of life of patients suffering from chronic locked posterior shoulder dislocation with associated articular defects. We hope that our findings will serve as a valuable resource and guide for orthopedic clinicians, ultimately empowering them to make informed decisions and provide optimal care for their patients.

## Figures and Tables

**Table 1:** Range of motion and total score in Constant’s scoring scale of the operated shoulder.

Patient	Flexion	Abduction	External Rotation	Internal Rotation	The Sum of Constant’s Scale
1	180	180	80	60	93
2	180	180	75	55	82
3	125	110	40	20	76
4	125	100	20	15	71
5	70	65	15	10	50
6	178	177	76	60	90
7	175	173	77	58	86
8	160	159	76	60	88
9	173	170	77	60	91
10	177	177	74	60	93
11	173	170	70	55	87
12	176	174	78	60	90
13	177	177	75	58	84
13	176	173	70	55	88
14	175	175	76	58	85
15	178	177	78	56	88
16	173	173	75	53	89
17	174	174	74	50	83
18	162	160	73	50	80
19	168	166	69	48	82
20	180	180	80	60	91
Average	164.52381	161.428571	68	50.5238095	84.14285714

## Data Availability

The data presented in this study are available on request from the corresponding author. The data are not publicly available due to privacy reasons.
